# Inclusion of Palm Kernel Cake in Supplements for Grazing Lambs on Growth Performance, Digestibility, Nitrogen Balance, Blood Metabolites and Meat Quality

**DOI:** 10.3390/ani16203306

**Published:** 2026-10-20

**Authors:** Bruna Yasnaia de Souza Oliveira, Kennyson Alves de Souza, Lopes César Mugabe, Fabiana Lana de Araújo, Rosani Matoso Silva, Mailin Vasconcelos dos Santos Lima, Emmanuel Emydio Gomes Pinheiro, Daniele Rebouças Santana Loures, Adriana Regina Bagaldo

**Affiliations:** 1 School of Veterinary Medicine and Animal Science, Universidade Federal da Bahia, Salvador 40170-110, Brazil; brunayasnaia@hotmail.com; 2 Center for Agricultural, Environmental and Biological Sciences, Universidade Federal do Recôncavo da Bahia, Cruz das Almas 44380-000, Brazildrloures@ufrb.edu.br (D.R.S.L.); 3 Faculty of Agronomic and Forestry Engineering, Universidade Zambeze, Mocuba 2403, Mozambique

**Keywords:** byproduct, pasture, performance, sheep

## Abstract

Palm kernel cake (PKC), a byproduct produced in various countries, has been studied for its potential use as animal feed. This study aimed to assess the effects of PKC as a feed supplement on Santa Inês lambs raised on pasture (*Panicum maximum* cv. Massai). Forty uncastrated lambs were included in a completely randomized design and allocated to four treatments consisting of feed supplements with different levels of PKC included (0.0, 150, 300, and 450 g/kg). The intake of dry matter, neutral detergent fiber, and non-fibrous carbohydrates and the time spent ruminating decreased linearly as the level of PKC increased. Digestibility of the ether extract improved, fecal levels and intake of nitrogen decreased, and microbial protein remained consistent with PKC inclusion. The final body weight, total weight gain, and average daily weight gain decreased quadratically with PKC inclusion levels. Marbling and meat color improved with the inclusion of PKC in the diet. Lambs grazing and supplemented with control rations exhibited superior performance compared to those consuming supplements with PKC content. Including PKC at levels up to 450 g/kg in feed supplement for grazing lambs may reduce feed intake and animal performance without affecting blood metabolites. However, it enhances meat redness, which may appeal to consumers. Its use may be advantageous during critical dry periods for the maintenance feeding of grazing animals.

## Simple Summary

Palm kernel cake, a byproduct of palm oil production, may be used as an alternative feed for sheep when pasture is limited. This study examined how different amounts of palm kernel cake affected the feed intake, health, growth, and meat quality of grazing Santa Inês lambs. Forty lambs were administered supplements with progressively increasing quantities of this ingredient while grazing on pasture. Lambs fed palm kernel cake ate less, spent less time chewing, and gained less weight. Their blood measurements and liver health were not affected, indicating no harmful effects. Most carcass characteristics were unchanged, but the meat had improved redness and marbling, indicating small amounts of fat within the meat. Although lambs grew best without palm kernel cake, it may help maintain grazing animals when usual feed sources are scarce, especially during the dry season.

## 1. Introduction

Rearing sheep on pasture presents notable advantages because of reduced costs related to infrastructure, equipment, skilled labor, and feed compared to the feedlot system. In grazing environments, human interference with animals’ social behavior is minimal, which has attracted interest from consumers willing to pay a premium, thus enhancing the value and quality of the final products [1]. For producers, supplementing grazing lambs with concentrated feed reduces the time required from finishing to slaughter and promotes faster weight gain. Additionally, supplementation can improve meat characteristics, such as tenderness and flavor, thereby increasing its appeal to consumers [2].

Corn and soybean meals have traditionally served as the primary feed components for ruminants. The annual fluctuations in the international market prices of these commodities have led researchers and producers to investigate alternative feeds or byproducts for inclusion in animal supplements. Among these alternatives, palm kernel, obtained from the fruit of the oil palm (*Elaeis guineensis* Jacq.), is of particular interest. This tree, native to West Africa, is primarily cultivated for palm oil production and is extensively grown in Asia, Africa, and South America, with its major producers including Indonesia, Malaysia, Thailand, Nigeria, and Colombia [3]. The palm fruit is pressed to extract palm oil, which is employed in both the food industry and biodiesel production.

Palm kernel cake (PKC), a byproduct of oil extraction, is notable for its high fiber content of 656.6 g/kg and ether extract of 9.7 g/kg, both on a dry matter basis [4,5]. The residual oil in the cake, along with its low acquisition cost in regions where palm oil is processed, has sparked interest in its use as animal feed. Nevertheless, the effects of PKC on ruminal fermentation remain unclear. Although research has been conducted on small ruminants in feedlots, data on the use of PKC under grazing conditions are limited [6,7,8].

The authors propose that palm kernel cake could be incorporated into the diet of grazing lambs to maintain satisfactory animal performance, health, and meat quality. This study aimed to assess the effects of PKC in supplements for Santa Ines lambs grazing Massai grass (*Panicum maximum* cv. massai) on intake, digestibility, growth performance, nitrogen balance, and meat quality.

## 2. Materials and Methods

### 2.1. Animals and Treatments

The experiment was conducted in the sheep unit at the Center for Agrarian, Environmental, and Biological Sciences (CCAAB) at the Federal University of Recôncavo da Bahia, located in Cruz das Almas, Bahia, Brazil.

Forty uncastrated Santa Inês lambs with an average age of 150 ± 15 days and mean weight of 25.2 ± 2.0 kg were assigned to four treatments in a completely randomized design. The treatments consisted of different levels of palm kernel cake inclusion in the supplement on a dry matter basis: 0, 150, 300, and 450 g/kg (Table 1 and Table 2). The supplements were formulated to achieve an average weight gain of 150 g/day [9]. The experimental period lasted 120 days, following a 15-day adaptation phase to the pasture area, facilities, and supplements.

The forty lambs were kept on one of two paddocks containing Massai grass (*Panicum maximun* cv. massai), sized 0.82 ha each, from 06:00 a.m. to 06:00 p.m., with access to drinking fountains. The lambs were transferred to another paddock according to forage availability. The residual height of the post-grazing pasture was between 25 and 30 cm to preserve grass growing points for new growth and regrowth. The optimized pasture utilization during the experimental period led to the forage availability above 1200 kg DM/ha (Table 3). Throughout the experiment, forage height was regularly measured at 10 random points in the paddock following animal grazing.

The lambs were fed the supplement individually at 06:00 a.m., at a rate of 10 g/kg of body weight, for approximately one hour, after which the animals were moved to the pasture. Refusals were collected and weighed daily to adjust the supplement intake.

The weights of the animals were recorded at the beginning and end of the experimental period following a 14 h fasting period to determine the initial and final body weights.

### 2.2. Samples and Analysis

Pasture samples were collected at 30-day intervals to assess the dry forage mass (kg/ha). This was achieved using the square method (0.50 m^2^), randomly performed at five locations in each paddock. Leaves, stalks, and dead material were cut at ground level, and two representative subsamples were collected from each paddock. One subsample was used to quantify the total forage mass of the pasture, while the other was used to determine the mass, leaf percentage, stem plus sheath, and dead material through manual forage separation. The samples were subsequently weighed and oven-dried at 55 °C for 24 h to ascertain the dry matter content, followed by milling through a 1 mm screen.

The potentially digestible DM was estimated using the equation proposed by Paulino et al. [10]: DMpd = 0.98 × (100 − NDF) + (NDF − iNDF). Here, DMpd represents potentially digestible DM, NDF denotes neutral detergent fiber, and iNDF indicates indigestible neutral detergent fiber (Table 3).

### 2.3. Diet Intake and Apparent Digestibility

Supplement refusals were collected and weighed to calculate the intake. The apparent digestibility and dry matter intake were estimated from fecal production. Fecal excretion was estimated by supplying 3 g of titanium dioxide per animal/day (external indicator) homogenized with the supplement for 12 consecutive days. From days 7 to 12, feces were collected directly from the animals’ rectum once a day at different times (07:00 a.m., 10:00 a.m., 01:00 p.m., 04:00 p.m., and 06:00 p.m.). Dry matter was determined after oven-drying at 55 °C for 24 h and then milling through a 1 mm screen, according to the AOAC Method 934.01 [11].

The titanium dioxide (Synth, Diadema, SP, Brasil) content in the feces was determined using atomic absorption spectrophotometry (Perkin-Elmer^®^, Überlingen, Germany), according to Myers et al. [12]. The fecal recovery of titanium dioxide was estimated based on the relationship between the amount of indicator excreted and ingested. Fecal production (FP) was calculated as FP (g/day) = indicator administered (g/day)/concentration of indicator in feces (g/kg DM).

On day one of the digestibility test, two people observed the forage intake of animals in each treatment and performed simulated grazing. Samples of pasture, similar to those consumed by the animals, were collected after one hour of observation.

The indigestible neutral detergent fiber (iNDF) of forage, supplement, refusals, and fecal samples was determined to estimate total intake. The samples were incubated in the rumen of four fistulated cattle for 288 h, and the residue was assumed to be indigestible [13]. Supplement intake was estimated by calculating the difference between the total concentration of each nutrient in the feed offered to the lambs and the amount in the refusals. The estimated pasture intake was calculated as PI = [(FP × iNDFF) − (SI × iNDFS)]/CIFO, where PI = pasture intake (kg/day); FP = fecal production (kg/day); iNDFF = iNDF concentration (kg/kg DM) in the feces; SI = supplement intake (kg/day); iNDFIS = iNDF in the supplement (kg/kg DM); and CIFO = iNDF forage (kg/kg DM) [14].

Forage, supplement, refusals, and fecal samples were pre-dried in a drying oven at 55 °C for 24 h and were ground in a Wiley knife mill (Tecnal, São Paulo, Brazil) through a 1 mm screen, according to method 934.01 [11]. The nitrogen concentration was determined using the Kjeldahl method 988.05 [11], and the organic matter was calculated using method 967.05 [11]. Ash content was measured through combustion at 550 °C for 16 h according to method 942.05 [11]. The ether extract content was determined using method 920.39 [11], the acid detergent fiber content was measured using method 973.18 [11], and the neutral detergent fiber content (corrected) was measured according to Mertens [15] using α-amylase. The lignin content was determined as described by Robertson and Van Soest [16]. Non-fibrous carbohydrates corrected for protein were calculated using the equation described by Hall [17]: NFC = 100 − [(CP − CPu + U) + EE + MM + NDFap]. Here, CP = crude protein, CPu = crude protein from urea, U = urea content, EE = ether extract, MM = mineral matter, and NDFap = neutral detergent fiber corrected for ash and crude protein.

### 2.4. Ingestive Behavior Activities

The lambs were individually observed from 07:00 a.m. to 06:00 p.m. after supplement feeding. The time spent on different activities was recorded by visual observation of all animals every 5 min by a trained team [18]. Data were collected to estimate the duration of grazing, ruminating, and idling periods. The sum of the replicates determined the total time spent on each of the activities.

### 2.5. Metabolic Parameters and Nitrogen Balance

During the digestibility assay, animals were confined in a cattle chute to facilitate the collection of urine samples, which were obtained approximately four hours after the intake of concentrated feed due to spontaneous urination. Urine was collected in disposable plastic cups, filtered, and a 10 mL aliquot was extracted. This aliquot was then diluted in 40 mL of 0.036 N sulfuric acid [19] and stored at −20 °C. Nitrogenous compounds were analyzed using the Kjeldahl method 960.52 [11]. Nitrogen retention (N-retained, g/day) was calculated as follows: N-retained = N ingested (g) − N in feces (g) − N in urine (g).

Urine volume was estimated based on urine creatinine content using a commercial kit (Labtest Diagnostica, S/A, MG, Lagoa Santa, Brazil) and spectrophotometer reading (600-PLUS, Tecnal, Piracicaba, São Paulo, Brazil), according to Santos et al. [20]. Purine derivatives (allantoin, uric acid, xanthine, and hypoxanthine) were estimated using colorimetric methods [21]. Total purine excretion was estimated as the sum of the amounts of allantoin, uric acid, xanthine, and hypoxanthine excreted in urine. The absorbed microbial purine (X, mmol/day) was calculated from the excretion of purine derivatives in urine (Y, mmol/day) according to Verbič et al. [22].

Blood samples were collected using jugular venipuncture with a needle engaged within Vacutainer^®^ tubes (Becton, Dickinson and Co, São Paulo, SP, Brazil) with 10 mL heparin. The samples were immediately sent to the laboratory and centrifuged at 2500 G for 15 min to obtain blood plasma, which was stored in 2 mL Eppendorf microtubes (Eppendorf, Hamburg, Germany) at −20 °C. Plasma concentrations of albumin, urea, total proteins, alanine aminotransferase, aspartate aminotransferase, and gamma-glutamyltransferase were determined using a colorimetric enzymatic method with commercial kits (Bioclin, Quibasa Química Básica Ltda., Belo Horizonte, MG, Brazil).

### 2.6. Meat Quality

At the end of the experiment, all 40 animals were slaughtered in a commercial slaughterhouse in accordance with the Brazilian Federal Inspection Regulations specified in the Brazilian Regulation of Industrial and Sanitary Inspection of Animal Products. After slaughter, the carcasses were identified, weighed, and chilled for 24 h at 4 °C. The longissimus dorsi muscle was removed from the left and right half-carcasses and stored at −20 °C. The pH and final temperature of the longissimus dorsi portion were measured immediately after slaughter and 24 h later.

The *Longissimus* muscle area was obtained from the muscle after a cross-section between the 12th and 13th ribs. Free analysis software, “ImageJ” (version 1.50), was used to measure the rib eye area, subcutaneous fat thickness, and percentage of marbling fat. The color of the longissimus dorsi was measured 30 h after slaughter according to the color system of the International Commission on Illumination (Commission Internationale de l’Eclairage − CIE). Color was measured on the L*, a*, and b* scales, where L* denotes luminosity, a* represents the green-to-red axis, and b* represents the blue-to-yellow axis. A Minolta colorimeter (Konica^®^ Minolta, Osaka, Japan)) with a D65 illuminant, 10° standard observer, and 8 mm aperture size was used. The mean values were calculated from six readings taken of the muscle slice areas for each sample.

Meat samples were thawed at 6 °C for 24 h and weighed; thawing losses were calculated as the percent difference between fresh and thawed weights. Subsequently, fresh meat samples were homogenized in a multiprocessor until a homogeneous mass was obtained for dry matter determination (DM). Crude protein was analyzed using the 992.15 method [11], ether extract was determined using the 960.39 method [11], moisture content was determined by oven-drying at 105 °C until a constant weight was obtained (method 950.46 B) [11], and ash content was measured through combustion at 550 °C for 16 h according to the 942.05 method [11]. The total carbohydrate content was estimated by subtracting the total fat, protein, ash, and moisture content from 100%.

### 2.7. Statistical Analyses

Statistical analyses were performed using the SAS OnDemand for Academics software (version 9.4) [23]. Data were analyzed based on a completely randomized design, according to the following model:

Y i = μ + T i + ε i

where μ is the overall mean, T i is the treatment effect, and ε i is the experimental error of the *i*-th group. Residual normality was verified using the Shapiro–Wilk test (via PROC UNIVARIATE), and homogeneity of variances was assessed using Levene’s test. Using PROC MIXED, the effects of palm kernel cake inclusion levels were evaluated via orthogonal polynomial contrasts, considering the linear and quadratic effects. The cubic effect was not considered when interpreting the results because of the lack of a biological basis for such a response. Regression equations were obtained using the PROC REG procedure. A significance level of *p* ≤ 0.05 was adopted.

## 3. Results

### 3.1. Nutrient Intake and Digestibility

The inclusion of palm kernel cake in the supplement decreased linearly with the intake (g/day) of total dry matter, supplement dry matter, organic matter, crude protein, neutral detergent fiber (NDF), and non-fiber carbohydrates (NFC) (*p* < 0.05).

The digestibilities of dry matter, organic matter, crude protein, neutral detergent fiber, non-fiber carbohydrate, and total digestible nutrient (TDN) content did not differ among the treatments. However, the inclusion of palm kernel cake resulted in a significant increase (*p* < 0.05) in ether extract (EE) digestibility (Table 4).

### 3.2. Ingestive Behavior Activities and Animal Performance

The duration of rumination decreased linearly as palm kernel cake levels increased (Table 5). According to the regression equation, the shortest grazing time, recorded at 460.5 min over 11 h, occurred with the inclusion of 201 g/kg of palm kernel cake in the supplement. Additionally, the minimum idle time, measured at 149 min over 11 h, was observed with 262 g/kg of palm kernel cake (*p* < 0.05).

The final body weight, total weight gain, and mean daily weight gain decreased quadratically with PKC inclusion levels (*p* < 0.05) (Table 5). The lowest final body weight (31.2 kg) was observed with a supplement containing 291 g/kg of palm kernel cake, according to the regression equation (Table 5). The minimum total weight gain, measured at 6.29 kg, was associated with 275 g/kg cake inclusion, whereas the lowest average daily weight gain, 80.18 g, was observed with 268 g/kg.

### 3.3. Nitrogen Balance, Urea Excretion, and Plasma Metabolites

The results indicate that nitrogen intake and fecal and urea excretion decreased linearly (*p* < 0.05) as the palm kernel cake inclusion level increased (Table 6). In contrast, the retained nitrogen (g/kg N intake), microbial crude protein production, and microbial efficiency (Pmic/kg TDN) did not differ significantly (*p* > 0.05; Table 6).

The serum total protein and globulin levels linearly decreased with increasing cake levels (*p* < 0.05); however, no effects were observed on serum albumin or urea concentrations (*p* > 0.05). The inclusion of palm kernel cake did not change the pattern of enzymatic activity of gamma-glutamyltransferase (GGT), alanine aminotransferase (ALT), and aspartate aminotransferase (AST) (*p* > 0.05) (Table 7).

### 3.4. Meat Analysis

Hot and cold carcass weights and cold carcass yield were similar among the treatments (*p* > 0.05). The maximum percentage of longissimus dorsi marbling fat (2.84%) was observed at 274 g/kg inclusion. The centesimal analysis of fresh meat obtained from the *Longissimus dorsi* muscle was similar among the different palm kernel cake levels (*p* > 0.05) (Table 8).

The pH parameters, temperature, and moisture loss during thawing were not influenced by the level of cake inclusion (*p* > 0.05, Table 8). The maximum red meat content (11.65) was observed at 422 g/kg palm cake inclusion in the supplement (*p* < 0.05).

## 4. Discussion

Palm kernel cake, a byproduct of oil extraction, is primarily used as fertilizer in environmental applications. Research on the use of palm kernel cake in small ruminants has predominantly focused on feedlot animals. In the present study, the authors assessed the feasibility of incorporating palm kernel cake into the diet of grazing sheep.

### 4.1. Feed Intake, Digestibility, Animal Performance, and Ingestive Behavior Activities

Several physiological mechanisms may have contributed to the reduction in total and supplement dry matter intake. Previous studies have indicated a decline in dry matter intake with increasing levels of PKC in the diet [6,7,24]. Vargas and Souza [25] conducted a meta-analysis on the inclusion of PKC in the diets of cattle, sheep, and goats and observed a decrease in dry matter intake across all species with PKC inclusion. Additionally, these authors noted that feedlot sheep consumed more neutral detergent fiber when PKC was incorporated into their diets. PKC also has a high lignin content, which increased the concentration in the supplement and may have contributed to reduced supplement intake. It may also have contributed to the trend toward reduced forage intake (*p* < 0.10).

In feedlot systems, mixing supplements and roughages into the total diet reduces feed selection. Conversely, in this study, sheep were grazing and received the supplement separately, which facilitated a reduction in feed intake. Palm kernel cake is also reported to be poorly accepted by small ruminants [24,26]. Observations indicated refusals of the diet containing the cake, leading to a decrease in the consumption of the diet with PKC. This reduction in DM intake was associated with decreased intake of organic matter, crude protein, NDF, and NFC. Although an increase in ether extract intake was anticipated, given that PKC is a lipid source, as typically observed in most studies [6,7,27], the decrease in supplement dry matter intake resulted in unchanged ether extract intake.

Contrary to our expectations, the inclusion of palm kernel cake in diets did not lead to a decrease in the digestibility of dry matter and crude protein, despite the higher levels of NDF, lignin, and neutral detergent insoluble protein. Low intake may have reduced the rumen passage rate and allowed more time for feed fermentation in the rumen without compromising the digestibility of diets containing PKC. In addition, Schettini et al. [28] explained that the compensation theory suggests an increase in feed utilization efficiency when feed is scarce within the physiological limits of satiety. This implies that energy compensation is associated with feed intake quantity.

Vargas and Souza [25] noted a trend of increased dry matter digestibility in sheep diets containing palm kernel cake, indicating that it provides nutrients that are efficiently digested in the rumen and supports ruminal fermentation as an energy and protein source [29]. Additionally, replacing up to 75% of corn with palm kernel cake in the diet did not alter the in vitro digestibility of dry matter and organic matter, nor did it affect methane production [30].

Palm kernel cake is characterized by a high saturated fatty acid content and enhanced ether extract digestibility. Specifically, it contains 37.8% lauric acid, 19.5% myristic acid, and 11.8% palmitic acid. These three fatty acids collectively constitute 70% of the total fatty acids [7], potentially accounting for the observed increase in ether extract digestibility when palm kernel cake was incorporated into the supplement (Table 4). Vargas and Souza [25] similarly reported an increase in ether extract digestibility in their research. Additionally, there was a trend (*p* = 0.0625) indicating a decrease in total digestible nutrient (TDN) intake, a response anticipated in other studies owing to the high neutral detergent fiber content of palm kernel cake.

The final body weight, total weight gain, and average daily gain recorded in this study were consistent with the recommended values for the Santa Inês breed. The average daily gain is influenced by feed intake, irrespective of the diet [31]. A reduction in feed intake led to a decrease in the final body weight, total weight gain, and average daily weight gain with the inclusion of palm kernel cake. The quadratic regression equation (Table 5) indicates that the increase in weight gain following the inclusion of 275 g/kg of palm kernel cake in the supplement could be attributed to the compensation theory. This theory posits that with a reduced amount of available food, its efficiency of use is enhanced within the physiological limits of satiety [28]. Similarly, Vargas and Souza [25] observed a reduction in weight gain in sheep fed diets containing PKC.

The feed intake and eating behaviors of ruminants differ from those of animals maintained in confinement. According to Cardoso et al. [32], the physical and chemical properties of supplements can influence pasture intake, thereby altering the ingestive behavior of animals. Consistent with this, a lower intake of neutral detergent fiber was associated with a reduction in rumination time (Table 5). Nevertheless, the total daily rumination time was not assessed, as the observation of ingestive behavior was limited to the daytime, while at night, the animals remained in the barn to simulate a typical farm routine.

### 4.2. Nitrogen Balance and Serum Metabolites

Excreted nitrogen acts as a pollutant and also necessitates energy expenditure by the animal, thus posing environmental and economic challenges. The excretion rates of nitrogen compounds in ruminant urine and feces are directly proportional to nitrogen intake [33]. This study’s field data indicate that animals on the control diet consumed 35.7% more nitrogen than those receiving 450 g/kg of palm kernel cake (Table 6). However, the blood urea concentration remained stable, suggesting that circulating blood urea was recycled in the rumen. This is because a reduction in protein content enhances the proportion of blood urea recycled into the rumen relative to the amount of urea excreted in the urine [34].

Research has shown that the inclusion level of PKC does not influence fecal or manure nitrogen, indicating that PKC is environmentally neutral with respect to nitrogen excretion [35]. This finding supports the incorporation of this approach into integrated management strategies aimed at reducing manure in ruminant production systems [35,36].

Total protein and globulin concentrations in the lambs’ blood decreased linearly as the inclusion level of palm kernel cake in the supplement increased; however, they remained within the normal ranges recommended for the sheep breed [37].

The serum concentrations of albumin, urea, gamma-glutamyl transferase, alanine aminotransferase, and aspartate aminotransferase remained stable, indicating that the palm kernel cake supplement did not induce liver disorders (Table 7).

### 4.3. Meat Quality

The *Longissimus* muscle area and subcutaneous fat thickness, which are indicative of tissue composition, were not influenced by the inclusion of palm kernel cake. Likewise, the chemical composition of the meat remained unaffected, likely because of the uniform age of the animals, which contributed to the similarity of the values (Table 8).

The final pH values, ranging from 5.53 to 5.66, aligned with the established standards for sheep meat, which are between 5.5 and 5.8 [38]. The pH level during rigor mortis development significantly affects muscle contraction, proteolysis, and protein denaturation, thereby influencing muscle fiber structure and, consequently, meat quality [39]. The observed standard pH and temperature values in this study suggest that all the evaluated diets facilitated optimal conditions for muscle glycogen storage, as evidenced by normal rigor mortis progression (Table 8). Thawing losses were consistent across treatments, ranging from 3.28% to 3.98%, which is within the typical range for sheep meat [40]. This outcome may be attributed to the similarity between the supplements provided during the experimental period.

The color of meat is influenced by several factors, including the protein pigment and fat content—which contribute to increased luminosity values [41]—as well as the pH decline during rigor mortis. The inclusion of palm kernel cake specifically altered the intensity of the meat’s red coloration without changing the L* and b* values or pH (Table 8). Thus, the observed increase in a* does not appear to be associated with changes in postmortem acidification or meat lightness. The increase in a* may be related to the presence of phenolic compounds with antioxidant potential in the palm kernel cake, given that these compounds have been identified in this co-product and have been shown to exhibit antioxidant activity [42]. De Castro et al. [43] suggested that differences in meat color associated with the use of palm products could be linked to the type of phenolic compound present in the palm kernel cake, considering its potential effect on inhibiting myoglobin oxidation and meat discoloration. Therefore, the presence of these compounds in palm kernel cake may have contributed to the greater red color intensity observed in the present study.

## 5. Conclusions

Including palm kernel cake at levels up to 450 g/kg in supplement for grazing lambs may reduce feed intake and animal performance without affecting blood metabolites. However, it enhances meat redness, which may appeal to consumers. Its use may be advantageous during critical dry periods for the maintenance feeding of grazing animals.

## Figures and Tables

**Table 1:** Chemical composition of the supplement ingredients.

Chemical Composition (g/kg DM)	Corn Grain	Soybean Meal	Palm Kernel Cake	*Panicum maximum* cv Massai
Dry matter (g/kg as fed)	871.8	880.9	904.8	307.9
Organic matter	921.2	908.2	931.2	922.3
Mineral matter	78.8	91.8	68.8	77.1
Crude protein	81.8	416.7	96.2	71.6
Ether extract	15.4	8.6	73.4	15.1
Neutral detergent fiber	85.1	101.3	697.7	695.1
NDIP ^1^ (g/kg CP)	89.7	22.0	469.0	460.9
Lignin	3.6	0.1	208.0	20.7
Cellulose	8.6	64.1	133.6	363.6
Hemicellulose	72.9	37.1	356.0	310.8
Non-fiber carbohydrates	738.6	381.4	63.7	144.3

^1^ Neutral detergent insoluble protein.

**Table 2:** Proportions and chemical composition of the supplements with palm kernel cake fed to grazing lambs.

Ingredients (g/kg DM)	Palm Kernel Cake Levels (g/kg DM)			
	0	150	300	450
Corn ground grain	620	455	350	220
Soybean meal	330	350	310	290
Palm kernel cake	-	145	290	440
Mineral supplement ^1^	20	20	20	20
Urea	30	30	30	30
	Chemical composition (g/kg DM)			
Dry matter (g/kg as fed)	831.2	836.2	840.6	845.4
Organic matter	920.9	922.0	924.0	925.0
Mineral matter	79.1	77.9	76.0	74.2
Crude protein	272.9	281.7	270.4	265.8
Ether extract	12.5	20.7	29.4	38.2
Neutral detergent fiber	86.3	175.4	263.6	355.1
NDIP ^2^ (g/kg CP)	62.9	116.6	174.3	232.5
Lignin	2.3	31.9	61.6	92.3
Cellulose	26.5	45.8	61.7	79.3
Hemicellulose	57.5	97.8	140.3	183.5
Non-fiber carbohydrates	551.6	446.6	363.0	268.9

^1^ Mineral salt composition (kg): 120 g calcium, 87 g phosphorus, 147 g sodium, 18 g sulfur, 590 mg copper, 40 mg cobalt, 20 mg chrome, 1800 mg iron, 80 mg iodine, 1300 mg manganese, 15 mg selenium, 3800 mg zinc, 300 mg molybdenum, and a maximum of 870 mg fluoride. The solubility of phosphorus in citric acid ranged from 2% to 95%. ^2^ Neutral detergent insoluble protein.

**Table 3:** Pasture characteristics during the experimental period.

Items	Period				Mean
	Dec/Jan	Jan/Feb	Feb/Mar	Mar/Apr	
Forage mass (kg/DM/ha)	2216	1322	3223	3476	2559
DMpd (kg/ha) ^1^	1281	818.1	1826	1965	1473
Forage height (cm)	62.4	44.5	100	73.0	70.0
Leaves (%) ^2^	41.8	71.1	61.2	33.6	51.9
Stem + sheath (%)	23.6	13.0	27.0	47.1	27.7
Dead matter (%)	34.5	15.8	11.8	19.4	20.4

^1^ Potentially digestible dry matter; ^2^ Dry matter basis.

**Table 4:** Nutrient intake and digestibility of grazing lambs supplemented with palm kernel cake.

Item	Palm Kernel Cake Levels (g/kg DM)					*p* Value ^2^	
	0	150	300	450			
	Intake (g/day)				SEM ^1^	L	Q
DM total ^3^	1105.4	828.9	943.0	502.2	75.2	0.0254	0.5905
DM pasture	899.4	642.9	818.0	389.7	78.0	0.0539	0.5529
DM supplement ^4^	206.0	186.0	125.0	112.5	14.2	0.0066	0.8763
Organic matter ^5^	1019.7	757.8	843.2	407.2	82.6	0.0265	0.5883
Crude protein ^6^	125.0	104.1	98.6	64.52	7.6	0.0064	0.6139
Ether extract	31.08	29.9	35.5	18.15	3.5	0.3160	0.2667
Neutral detergent fiber ^7^	620.4	447.9	545.7	238.0	56.0	0.0378	0.5121
Non-fiber carbohydrates ^8^	243.4	175.8	163.4	86.47	17.3	0.0114	0.9626
Total digestible nutrients	687.3	512.8	590.1	273.4	67.0	0.0625	0.5820
	Digestibility (g/kg)						
Dry matter	666	652	639	682	13.2	0.7657	0.3282
Organic matter	694	708	659	708	13.1	0.9577	0.5314
Crude protein	707	744	706	786	12.2	0.0552	0.3335
Ether extract ^9^	632	707	764	776	22.1	0.0038	0.3742
Neutral detergent fiber	700	734	682	706	15.8	0.8251	0.8883
Non-fiber carbohydrates	635	527	545	503	23.6	0.0911	0.4739
Total digestible nutrients	622	617	595	589	20.5	0.6147	0.9810

^1^ Standard error of the mean; ^2^ L = linear effect, Q = quadratic effect. Significance at *p* < 0.05. ^3^ L: Y = 1095.06 − 1.0819x (R^2^ = 0.2490); ^4^ L: Y = 208.89 − 0.2306x (R^2^ = 0.3948); ^5^ L: Y = 999.52 − 0.9919x (R^2^ = 0.2413); ^6^ L: Y = 125.87 − 0.1221x (R^2^ = 0.3749); ^7^ L: Y = 617.25 − 0.668x (R^2^ = 0.2116); ^8^ L: Y = 239.34 − 3.18x; ^9^ L: Y = 641.90 + 0.3267x (R^2^ = 0.5713).

**Table 5:** Intake behavior and performance of grazing lambs supplemented with palm kernel cake.

Item	Palm Kernel Cake Levels (g/kg DM)				SEM ^1^	*p* Value ^2^	
	0	150	300	450		L	Q
Feeding ^3^ (mins/11 h)	486	449	477	488	4.87	0.3517	0.0108
Rumination ^4^ (mins/11 h)	71	63	47	43	3.28	0.0003	0.6931
Idleness ^5^ (mins/11 h)	103	148	136	130	4.59	0.0683	0.0030
Initial body weight (kg)	26.02	25.28	24.88	24.72	0.11	0.2572	0.7304
Final body weight (kg) ^6^	36.06	32.12	31.40	32.48	0.66	0.0921	0.0084
Total weight gain (kg) ^7^	10.04	6.84	6.52	7.76	0.41	0.0487	0.0078
Average daily weight gain (g) ^8^	83	57	54	64	4.48	0.0487	0.0078

^1^ Standard error of the mean; ^2^ L = linear effect, Q = quadratic effect. ^3^ Q: Y = 0.00051x^2^ − 0.2082x + 481.53 (R^2^ = 0.1640); ^4^ L: Y = 70.95 − 0.067x (R^2^ = 0.2998); ^5^ L: Y = 108.85 + 0.2940x − 0.00056x^2^ (R^2^ = 0.2563); ^6^ Q: Y = 0.000056x^2^ − 0.03270x + 35.9905 (R^2^ = 0.1747); ^7^ Q: Y = 0.000049x^2^ − 0.02696x + 9.9975 (R^2^ = 0.2510); ^8^ Q: Y = 83.13 − 0.022x + 0.000041x^2^.

**Table 6:** Nitrogen balance, urea excretion, and microbial efficiency in grazing lambs supplemented with palm kernel cake.

Nitrogen	Palm Kernel Cake Levels (g/kg DM)					*p* Value^2^	
	0	15	30	45	SEM ^1^	L	Q
N-Intake (g/day) ^3^	19.99	16.66	15.78	12.85	0.47	0.0470	0.9306
N faecal (g/day) ^4^	5.63	4.30	4.19	2.64	0.36	0.0063	0.0583
N Urinary(g/day)	0.98	0.67	0.43	0.44	0.06	0.1403	0.1205
N-Retain (g/day)	13.38	11.68	11.14	9.54	0.22	0.1677	0.9819
Urea (g/day) ^5^	1.04	1.18	0.71	0.73	0.06	0.0127	0.0524
Pmic/kgTDN	28.99	36.38	38.14	41.54	2.33	0.0571	0.1471
CP microbial (g/day)	183.3	170.9	237.9	201.1	1.24	0.7527	0.8241

^1^ SEM = mean standard error; ^2^ L = Significance for linear effect; Q = Significance for quadratic effect; significant probability at the 5% level. ^3^ L: Y = 19.6730 − 0.01487x (R^2^ = 0.2224); ^4^ L: Y = 5.5550 − 0.00605x (R^2^ = 0.4054); ^5^ L: Y = 1.12022 − 0.00923x (R^2^ = 0.3221).

**Table 7:** Serum metabolite concentrations in grazing lambs supplemented with palm kernel cake.

Item	Palm Kernel Cake Levels (g/kg DM)				SEM ^1^	*p*-Value ^2^	
	0.0	150	300	450		L	Q
Total protein (g/dL) ^3^	6.76	6.80	5.82	6.09	0.15	0.0280	0.6970
Albumin (g/dL)	1.55	1.66	1.52	1.58	0.06	0.9144	0.8744
Globulin (g/dL) ^4^	5.27	5.18	4.28	4.51	0.15	0.0163	0.5248
Albumin:Globulin	0.29	0.32	0.37	0.36	0.01	0.1163	0.2676
Urea (mg/dL)	32.73	33.77	35.57	36.17	0.98	0.1853	0.4121
GGT (UI)	107.34	100.70	88.88	94.71	9.37	0.5823	0.7537
ALT (UI)	17.50	20.48	16.44	14.05	1.25	0.2179	0.3444
AST (UI)	9.31	8.01	10.73	15.65	1.75	0.0951	0.2673

^1^ Standard error of the mean; ^2^ L = linear effect, Q = quadratic effect; Significance at *p* < 0.05. ^3^ L^2^: Y = 6.80561 − 0.001939x (R^2^ = 0.1191); ^4^ L^2^: Y = 5.3170 − 0.00218x (R^2^ = 0.1488).

**Table 8:** Analysis of the *Longissimus dorsi* of grazing lambs supplemented with palm kernel cake.

	Palm Kernel Cake Levels (g/kg DM)				SEM ^1^	*p*-Value ^2^	
Item	0	150	300	450		L	Q
Carcass Yield							
Hot carcass weight (kg)	14.5	14.0	13.1	13.7	0.79	0.3662	0.4586
Cold carcass weight (kg)	14.4	13.9	13.0	13.7	0.79	0.3836	0.4383
Cold carcass yield (%)	39.1	40.7	38.6	39.2	0.95	0.7264	0.6283
Tissue proportion							
Muscle (%)	52.1	53.9	53.2	52.3	0.86	0.9656	0.4575
Bone (%)	32.8	33.8	34.7	34.7	1.03	0.4792	0.8341
Fat (%)	12.1	11.8	11.6	11.1	0.33	0.5583	0.8401
Loin eye area (cm^2^)	8.7	9.8	9.2	8.6	0.37	0.5186	0.1960
Marbling (%) ^3^	1.9	2.0	2.9	1.9	0.23	0.1965	0.0296
Centesimal analysis							
Humidity (%NM) ^4^	74.1	72.7	74.4	73.8	0.33	0.5056	0.3918
Fat (%NM)	2.9	2.7	2.4	3.0	0.15	0.3885	0.0818
Crude protein (%NM)	20.9	22.4	21.1	21.3	0.28	0.9638	0.4723
Total carbohydrate (%NM)	1.92	1.02	1.50	1.8	0.21	0.5558	0.6595
SFT (mm) ^5^	3.0	3.0	2.5	2.5	0.17	0.9244	0.8652
Ash (%NM)	1.83	1.51	1.20	1.27	0.11	0.0316	0.3314
Meat quality parameters							
Initial pH	6.0	5.9	5.9	6.0	0.035	0.7616	0.6118
pH/min drop	0.01	0.01	0.01	0.02	0.002	0.5672	0.1632
Final pH	5.6	5.7	5.6	5.6	0.036	0.3250	0.2072
Initial T (°C)	27.8	28.0	28.2	28.2	0.085	0.7480	0.9498
Final T (°C)	18.9	18.9	19.5	19.5	0.149	0.2024	0.3227
Defrost moisture loss (g)	3.58	3.98	3.28	3.65	0.186	0.9097	0.9792
Color							
L*	42.8	43.1	41.3	43.5	0.500	0.9747	0.6393
a* ^6^	9.6	11.2	11.2	11.4	0.296	0.0091	0.0230
b*	12.3	12.8	11.9	13.2	0.237	0.4688	0.5757

^1^ Standard error of the mean. ^2^ L = linear effect, Q = quadratic effect. Significance at *p* < 0.05 ^3^ Q: Y = − 0.0025 + 0.1371x + 0.9624x^2^. ^4^ NM = natural matter. ^5^ SFT: subcutaneous fat thickness. ^6^ Q: Y = − 0.0011 + 0.0946x + 9.6549x^2^.

## Data Availability

The data presented in this study are available on request from the corresponding author. (Data are not publicly available due to restrictions on the research group).
